# Recent Advances in Intelligent Algorithms for Fault Detection and Diagnosis

**DOI:** 10.3390/s24082656

**Published:** 2024-04-22

**Authors:** Paolo Mercorelli

**Affiliations:** Institute for Production Technology and Systems, Leuphana University of Lueneburg, Universitaetsallee 1, D-21335 Lueneburg, Germany; paolo.mercorelli@leuphana.de

**Keywords:** fault detection techniques, high impedance fault, modeling, fault location techniques, literature review

## Abstract

Fault-finding diagnostics is a model-driven approach that identifies a system’s malfunctioning portion. It uses residual generators to identify faults, and various methods like isolation techniques and structural analysis are used. However, diagnostic equipment doesn’t measure the remaining signal-to-noise ratio. Residual selection identifies fault-detecting generators. Fault detective diagnostic (FDD) approaches have been investigated and implemented for various industrial processes. However, industrial operations make it difficult to implement FDD techniques. To bridge the gap between theoretical methodologies and implementations, hybrid approaches and intelligent procedures are needed. Future research should focus on improving fault prognosis, allowing for accurate prediction of process failures and avoiding safety hazards. Real-time and comprehensive FDD strategies should be implemented in the age of big data.

## 1. Introduction

### 1.1. Model-Based Diagnostics

The diagnostics for fault finding may detect malfunctions in a system and identify the offending part. Model-based and data-driven approaches are used in diagnostic procedures. By comparing model residuals to actual system data, model-based diagnostics may identify problems. The model-based design of diagnostic systems is founded on a library of residual generators, each responding to a certain class of system problems. Using triggered residuals, a fault isolation technique generates diagnostic possibilities or fault hypotheses. Finding residual generator sets with high fault solubility may be possible using efficient approaches based on structural analysis. These methods take into account only perfect breakdowns for each residual generator. That means the remaining signal-to-noise ratio isn’t measured by diagnostic equipment [1]. The defect affects all residuals in the figure, but only a subset of them deviates considerably from their expected behavior. Residual selection aims to identify generators that can detect and isolate faults. To discover these sets, researchers have created tools like the Binary Integer Linear Programming (BILP) algorithm and the greedy search technique [1,2]. The fault detection capabilities of each potential residual generator are considered equivalent in these pieces. The proposed residual selection approach is quantitatively based on the performance of the residuals. Model correctness and measurement noise on diagnostic efficacy may be simulated using data-driven methodologies using actual measurement data.

### 1.2. Data-Driven Diagnostics

Researchers have proposed different classification schemes for defect diagnostics based on collected data. The lack of models is not a prerequisite for data-driven flaw discovery. To perform well, data-driven classifiers need a large amount of relevant training data. Collecting bad information that accurately represents each failure scenario takes a lot of time and effort. As a result, the available training data and the number of possible fault realizations are not the same [3,4].

With this data, a data-driven classifier cannot be trained to reliably detect and isolate errors. A system model with tunable fault sensitivity may be used to design fault-tolerant residual generators. The Kalman filter and particle filter are two techniques for creating residual generators. Occasionally, Computer-Aided Design (CAD) software can generate residual generator C or Matlab code automatically. Consider that the number of residual generators may exceed that of the sensors. When the training data are not generic enough to cover all possible defect realizations, the structural information of model-based residual generators may be used to build a diagnostic system that can still locate the source of the issue. Feature selection in machine learning is a special case of the residual selection issue. Overfitting and complexity in data-driven models may be greatly reduced with careful feature selection. Using data-driven feature selection to locate model-based residual generators is one example of how combining model-based and data-driven approaches may improve problem detection and isolation.

### 1.3. Intelligent Diagnostics Process

The intelligent diagnostics process includes monitoring a device’s operation, capturing signals using sensors that express operating parameters, and logically comparing the received data with a reference value. Diagnostics are carried out in the real operation mode or during a test impact on the object [5].

In the process of intelligent diagnostics, you can obtain a result about the health of the object. A more difficult task is to locate the fault. This is achieved by special methods and troubleshooting methods implemented by diagnostic algorithms.

### 1.4. Troubleshooting Methods

For troubleshooting, the following basic methods for optimizing the check programs of the diagnosed system are used:

Method of sequential, functional analysis: This is one of the very first ways to monitor performance and troubleshoot. This method is based on the determination of the main functions of the device and the consistent control over the performance of these functions employing tracking of the values of the parameters that are affected by the functions. If the parameter definition tolerance is out of range, troubleshooting starts. The search begins “from the output of the device”, sequentially going through the nodes of the device until the parameters at the node in question are within the tolerance limit.

Method of half partition: This method is used in mechanisms with elements connected in series. The essence of the method is to divide the components of the device into two parts, followed by an analysis of the output parameters of each part. Then, the part in which the parameter value is out of tolerance is selected and again divided into two parts. The division continues until a faulty node is found.

Time-probability method: Using this method, the sanity check begins with the nodes most prone to breakage, on which the minimum amount of check time is spent. Thanks to this technique, the minimum required number of search procedures is reduced, reducing the total time for diagnosing the system. The method is used in systems where nodes operate independently of each other. That is, the information obtained when checking one node is not considered when checking another.

Engineering method: This method is used in the development of diagnostic algorithms. The engineering method is based on the calculation of the preference function, which is selected based on the initial data and diagnostic tasks. The resulting algorithms can be applied to troubleshooting and assessing the performance of nodes.

Information method: Constructing a fault algorithm based on the information method allows you to select the minimum number of controlled parameters and determine the sequence in which you need to control them.

The method is based on the hierarchical principle. In this method, the functional elements of the device are divided into several groups, while the output parameters of each group are combined at one point with a fault indicator. When a malfunction occurs, it is easy to determine the health of a group of nodes using an indicator.

### 1.5. Backpropagation Algorithm

A backpropagation algorithm is known, which makes it possible to identify and classify a fault. In the learning process, the neural network input receives data from the training sample when adjusting the weight coefficients of synaptic connections. When using the backpropagation algorithm, the error signal at the neural network’s output propagates in the opposite direction, with subsequent adjustment of the synaptic weights of the neural network to achieve the minimum output error. The trained network can generalize, i.e., it can provide a statistically correct response to input signals that belong to the training data class but are not used either in training or testing. The disadvantages of this algorithm include large time costs.

To speed up the learning process, an elastic propagation algorithm was developed. In this algorithm, only the signs of derivatives of a particular case are used to adjust the weight coefficients. A certain rule is used, according to which the weight correction value is calculated. If the derivative changes from a plus to a minus sign, then the error increases, and the weight decreases. If the sign changes from minus to plus, then the weight increases.

It is proposed to create a method for intelligent equipment diagnostics based on the method of sequential, functional analysis and the error backpropagation algorithm. The essence of the method is to determine the type of probabilistic distribution of the signal received from the equipment using sensors.

## 2. Materials and Methods

### 2.1. Introduction

The FDD for many different processes has attracted much attention from various industrial sectors and academia over several decades [6]. This is because the FDD offers many substantial benefits, such as reducing the costs related to the process or the product, as well as improving quality and productivity. In particular, FDD has been a major factor in several processes that are a part of industrial engineering. These processes include the fabrication of semiconductors, chemical engineering, and software engineering. Therefore, for the efficient and effective operation of processes, there is a growing demand for the efficient detection and diagnosis of suspicious faults to prevent a deterioration of the process, which could ultimately lead to a decrease in product yield or process throughput. This is because the process could cause a deterioration in either of these metrics.

### 2.2. Approaches to FDD

In most cases, the FDD work is carried out following some process and equipment data that are measured by instruments. This serves as a primary approach for the supervision of the processes (i.e., sensors). Also, to this day, many applications and research on the detection of a faulty process (or variable) have considered a variety of competent analytical approaches, and those on the diagnosis, isolation, and identification of causes of a process anomaly after the detection has adopted various mathematical or probabilistic models. Also, to this day, many applications and research on detecting a faulty process (or variable) have considered a variety of competent analytical approaches. Both theoretical and experimental research have been conducted on FDD approaches for various industrial processes on multiple occasions. Data-driven, model-based, and knowledge-based approaches can be categorized according to how they obtain their information. Particularly, data-driven and model-based approaches require very little modeling effort and prior knowledge of the process (or variables) of interest. These approaches have made a huge impact on FDD for industrial processes thanks to their relative simplicity and effectiveness for process FDD, and they require very little modeling effort. The online real-time FDD is also another key issue in the present process monitoring sector. This is especially true in certain industries containing dangerous processes, such as large-scale chemical processes, where it is necessary to monitor them in real-time. By identifying anomalous symptoms in the early phases of the formation of process flaws using the FDD tool, it is possible to boost the process’s efficiency as well as its level of safety. However, as a result of the feedback masking effects, it can be extremely challenging to identify errors in the control loops of chemical processes. It is usual practice to contemplate the creation of a bank of FDD models that can differentiate between faulty state and normal status to overcome this issue.

### 2.3. Paper Structure

The remaining portions of the paper are structured as follows: In Section 1, the terminology that is utilized in the field of process monitoring, the notion of process monitoring and FDD, as well as the industrial applications of FDD are discussed. The data-driven FDD approaches and the applications of those methods are presented in Section 2. In Section 3, you will find a discussion of the model-based FDD techniques as well as their applications. Section 4 provides for the conclusions and after that, some findings and suggestions for further research are offered.

## 3. Discussion and Analysis of the Existing Literature

### 3.1. Discussion

#### 3.1.1. Fault Detection and Diagnosis Techniques

**Figure 1:**
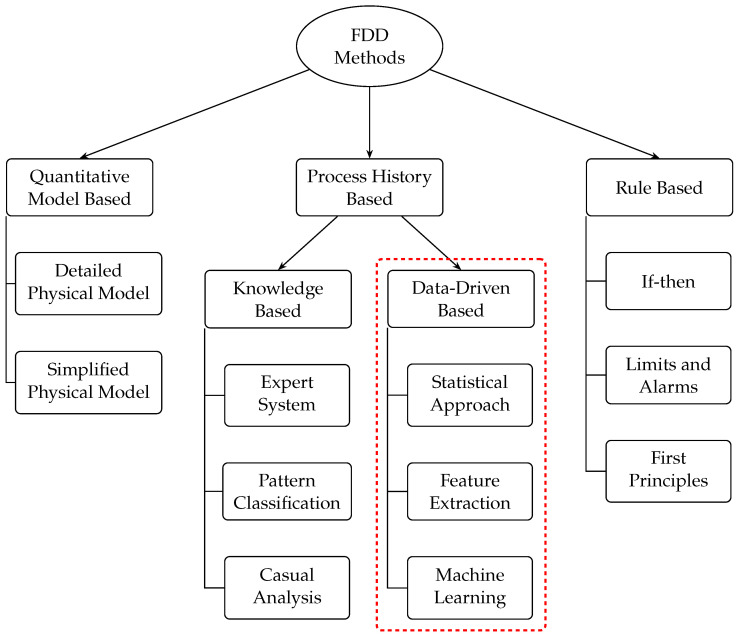
Fault Detection Techniques.

When an industrial process presents deviations in its parameters, its outputs generally do not correspond to normal values within the operating range [1,7,8]. These deviations could be caused by damage or malfunction of the devices involved in said process. Undesired outputs are considered failures, that is, deviations from the normal behavior of the plant or its instrumentation. Much research has been carried out for fault detection and diagnosis, through the application of mathematical algorithms and signal models [5,9]. The first investigations were carried out at the beginning of the 70s, based on the design of observers and the use of filters. Later the principle of analytical redundancy was developed [2,10,11]. Subsequently, for the first time, a specialized monitoring system was implemented for the detection and diagnosis of failures in a nuclear plant [5,12,13]. In recent years, promising techniques have been investigated and applied, such as parity space [14,15,16], fuzzy logic, and neural networks [17,18,19], among others. In Figure 1 the fault detection techniques are shown and in particular the Data-Drive Based method are underlined in a red color.

#### 3.1.2. Implementation and Challenges

Many of the methods developed present the use of a mathematical model of the process, which in some cases is not possible to obtain easily, and also include a mathematical analysis that presents considerable complexity [20,21,22]. In other methods, external devices are required whose cost, size, or weight limits their use. An alternative approach for fault detection and diagnosis is referred to the design of fault models and error analysis, where possible faults are represented by mathematical models and patterns that are capable of recognizing the occurrence of a fault at a given time [15,23,24]. In a similar way to this approach, the technique of robust transition structures has been proposed, which is effective for the control of processes with multiple regimes or operating points [4,25,26]. Initially, the error is defined as the absolute value between the process output and the process models in normal operation and each of the failures. Then a permissible error threshold is found to consider the process free of failure, based on the historical and heuristic knowledge of the process.

**Figure 2:**
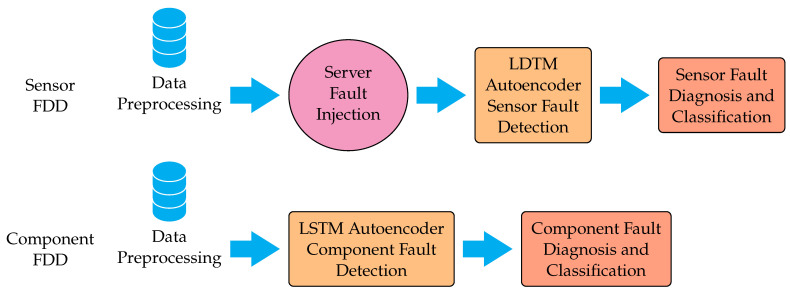
Challenges in Fault Detection Process.

The failure detection and diagnosis process is carried out in two stages: the first stage consists of a first online verification [27,28,29]. Fault detection is carried out by measuring the error values. Depending on this error, a code is generated that indicates if there is a process imbalance. From this code, the existence or not of a fault is verified. The fault code is a binary of n bits (it is made up of zeros and ones); depending on the location of the ones, it will warn the detection system that an abnormality exists [8,30,31]. The number n of bits of the code will depend on the number of fault models detected. To simulate the failures of the instrumentation used, such as transmitters and valves, simulated deviations are introduced in their parameters as additive disturbances of step type and an amplitude of 10% in the instrument parameters, such as failures in the transmitter span (FST), Transmitter Span Dead Zone Failure (ZMST), Transmitter Zero Calibration Failure (FCCT), Transmitter Zero Dead Zone Failure (FZMCT), Valve Span Failure (FSV), and failure in the valve span dead zone (FZMSV) [7,25,32,33]. After simulating the deviations in the parameters and with the specified amplitudes, the outputs of each of the previously found models are obtained, measuring the absolute value of the error and thus obtaining the failure code to determine if there is any abnormality in the process [34,35,36]. If the failures occur over some variations in values in which they have been modeled, there are offline models, and they will will only be used if the main models fail to detect the origin of the existing failure, according to the failure code returned by the system’s detection [7,21,37,38,39,40,41]. Disturbances or noises that are not caused by instrumentation failures, but by variations and/or noises inherent to the process, were also considered to verify the output and robustness of the proposed method. In Figure 2 the challenges in fault detection process are summarized.

#### 3.1.3. Set-Point Changes and Solutions

For a change in the set-point or reference value, two possible solutions are considered: Eliminate the change effect, where, for this purpose, the transfer function representative of the set-point variation is designed. This transfer function represents the relationship between the control signal and the change in the set-point and is subtracted from the value of the control signal when the change occurs [27,28,35,42,43]. On the other hand, the design of a second bank of calculated models, including the variation in the set point, could be considered: A variation of 10% of the original reference value is taken as an example, and the models are calculated in the same way as the previous case [22,44,45,46]. A new alternative for fault detection and diagnosis was proposed based on and taking advantage of the characteristics of a method originally used for the control of processes with different operating regimes [23,27,47,48,49]. One of the advantages of the proposed method is the use of only the input signal (control signal) and the output of the process to obtain the models. This is in addition to being a technique that does not require too much mathematical complexity, as only available signals and the use of parametric identification techniques are used; that is, it is therefore oriented towards a practical sense that can be implemented in any type of process [10,11,13,19]. In other words, it is possible to demonstrate that any abnormality in the process can be represented by a mathematical model through identification systems, based, of course, on the history of the process that is being studied, which helps to fulfill the initial objective of the AI detection [13,50,51,52]. The analysis was carried out for failures that are present in the process that is being controlled. The execution of all the models is avoided by using the second stage offline because it will only go to it if it is required. This means that it will only go to it if it is not feasible to locate the location of the failure that happened using the main models. In addition to this, the execution of all the models is avoided [22,45,53]. It is taken into consideration that the failures that may occur close to the values that were initially modeled do occur. As a higher number of failures are taken into consideration, the number of models will expand. This may be an annoyance if new failures occur; however, it is also feasible to regroup the models according to the characteristics that they exhibit [40,47,54,55]. In addition to the benefits that have already been described, this strategy also has the advantage of being able to take into consideration a wider range of values for potential failures, even when using offline models [2,11,42,56]. A degree of dependability in the findings may also be achieved by the use of the Student’s *t*-test, which determines whether or not the output of the process at a certain point belongs to the many failure models that are already in existence. Making use of non-parametric models for the process models and the failures that are associated with them is one way that the approach that is now being used might be improved for future study. Finding a model of the control dynamics is advised to limit the number of model banks that are used. This is because the control dynamics change depending on the reference value [29,32,39,40,46].

#### 3.1.4. Future Directions and Considerations

One further thing that should be taken into consideration for future research is the incorporation of non-linear valves or transmitters that have a temporal delay and the investigation of their behavior. Taking into consideration the aforementioned, the study in question suggests using the approach of resilient transition structure for defect identification and diagnostics [36,55,57,58]. Transition structures are recommended as an option for defect detection because of the features of the technique and the systems to which it is applied (change in the operating regime). This is because, in this particular instance, changes in the characteristics of the process are also given. These are taken into consideration throughout the process of designing the approach, which includes a collection of fault models that are indicative of the faults and allow for the estimation of whether or not the transition structures are feasible for the detection and diagnosis of faults.

#### 3.1.5. Application in Industries

Tradition in business has placed a premium on quality, efficiency, and cost-effectiveness. As a result of technological advancements in the last decade, finding and isolating problems has become an increasingly pressing issue for many industries. These advancements make it possible to conduct smart, low-cost condition measurements of equipment and monitor medium-scale transportation systems. Data from sensors placed at key nodes may provide a wealth of information about the system’s health as a whole.

If appropriately examined, this information may guide an operator toward actions that improve an asset, system, process, or plant uptime, such as reducing the frequency of unplanned downtime and increasing throughput [2]. Authentic datasets are few, making developing and evaluating fault detection and isolation algorithms challenging.

#### 3.1.6. Testing Approaches

**Figure 3:**
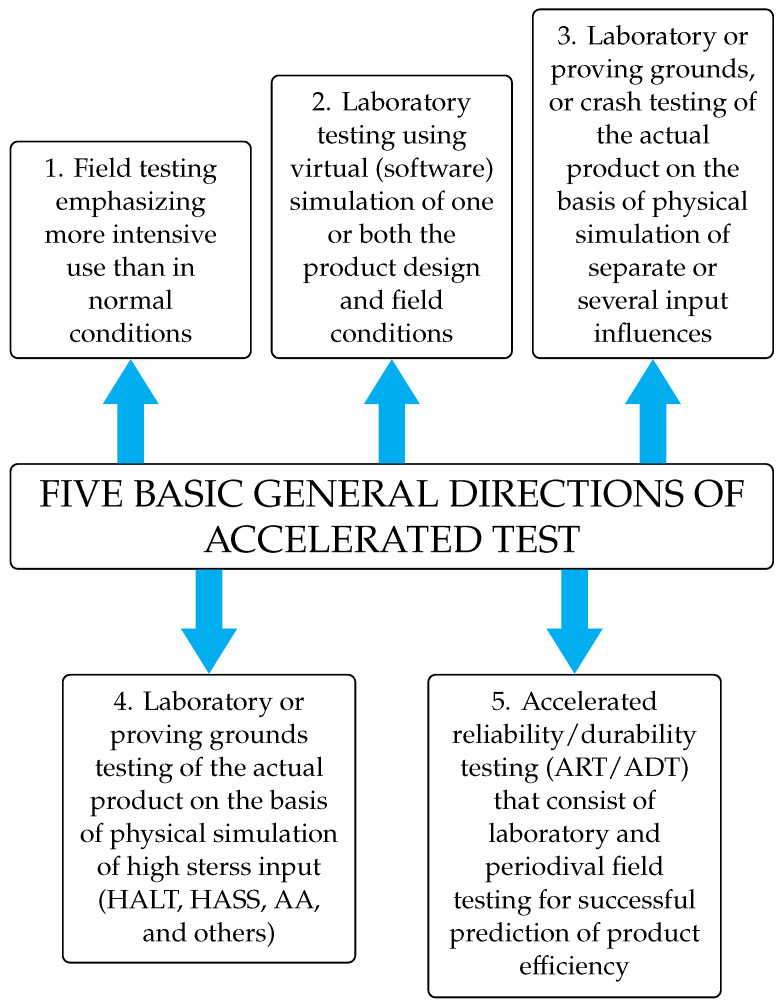
Testing Approaches.

It is harder to conduct representative testing on a gasoline system due to the high cost, low repeatability, and extensive time commitment required to detect even minor degradation. It is possible to approach this issue from three different angles. The most common technique is accelerated testing, which involves subjecting a part to more stress by increasing its workload or by making it out of less sturdy materials. Second, “seeded fault” testing allows for the components to be machined in a way that simulates the deteriorated state. The second choice just records a fleeting instant during the deterioration process, but it may be performed often to accumulate more effect. Third, by replicating certain degradation modes, such as by switching out a filter for a valve and imitating a clogged filter failure mode by gradually closing the valve. The third choice represents the medium position that this research adopts. Some sectors, like aerospace, have a substantial challenge in optimizing sensor sets in terms of defect detection accuracy since each additional sensor reduces asset availability [3,14]. Because there is no universally accepted approach, original equipment manufacturers (OEMs) have to develop application- and system-specific methodologies and tools to establish the minimum number of sensors required to meet failure detection and isolation (FDI) requirements. It was shown how a quantitative model-based approach to instrumentation optimization might be implemented using the same fuel system testbed. In Figure 3 the testing approaches are summarized.

### 3.2. Analysis

This study extends earlier work; it investigates the differences in the FDI accuracy achieved by using optimum vs. non-optimized sensor set solutions.

The research on defect detection approaches may be loosely split into two camps, model-based and data-driven, depending on the a priori process knowledge needed. The model-based approaches need extensive knowledge of physics underpinning the process. Some examples of approaches that are based on the models described in the thesis include parameter estimation methods, parity relation methods, and fault tree methods. Data-driven methods often presume access to a large quantity of historical process data. These methods enable the diagnostic system to be presented with a priori knowledge of the historical process data through feature extraction. Qualitative feature extraction methods include expert systems and qualitative trend analysis. There are also both statistical and non-statistical techniques for extracting quantitative features.

Quantitative feature extraction strategies include PLS/PCA, ANNs (artificial neural networks), and SVMs (support vector machines) [4,6,14,17,23]. We assume without further investigation that there exists both the measurement data collected from nominal and problematic system behavior and a model used for residual generation. Model information is used to identify groups of residual generators that are sensitive to the intended faults but insensitive to unwanted ones, hence achieving fault isolability characteristics. Data from both the model analysis and the training data are used to select residual sets for fault identification and isolation. After additional processing, the residuals are used to calculate the test amounts. One frequent approach to establishing the test quantities is to choose the “best” residual generators based on the structural analysis results. The diagnostic system is intended to consider the correlation between multiple residual generators to boost detection performance without resorting to additional residual generators.

In industrial settings, diagnostic systems face a significant challenge in evaluating defects based on limited data. While there is always a need for insightful data, working in the industry sometimes means dealing with incomplete or inaccurate datasets and shaky measurements. The complexity of this issue grows in tandem with the level of sophistication of the data transmission technique and the breadth of the process database. These problems may be fixed in several ways, from eliminating missing data to making informed predictions. Other machine learning methods can handle missing data without further processing, whereas most others cannot. When it comes to imperfect data, BBN can manage it with ease. It uses probabilities to assess the degree of uncertainty and, on occasion, uses expectation maximization techniques to learn parameters when there is some missing data in the data set.

It is essential in machine learning that models can be generalized. Overfitting, inability to generalize, and a large number of false positives, when given with a new observation, are all possible outcomes of a model that is too complex about the issue we are attempting to describe. Because of this, pruning is used in methods such as decision trees and neural networks. The clipping method seeks to avoid overtraining by cutting off the workouts suddenly. Further, most of these methods are strictly controlled, expert-led methodologies used by medical practitioners. A significant limitation of the controlled methods is that the model can only make assumptions about the kind of issues that have been encountered. Some models may use ambiguity rejection or distance rejection to account for the fact that they now know less about the system and its history.

Unfortunately, not all machine learning systems directly use the numerical data collected by sensors during the diagnostic process. Most machine learning methods, including SVMs and ANNs, need continuous data to operate. While continuous variables are used in certain methods, such as decision trees and BBN, others use discrete ones. Decision trees may now include numeric target variables in C4.5. Discrete variables are used by certain algorithms like ID3 and CART [17,23], whereas continuous variables are transformed into intervals by others. Although continuous random variables with Gaussian distributions are permitted in BBNs, discrete random variables are the norm. The fuzzy neural network is the only method capable of processing various data types (numeric and character). The result is a nebulous assemblage of both quantitative and qualitative information. The diagnostic process may be improved with the operator’s help if he or she can make the most of their knowledge and experience. One option is to create a model that can process numeric and textual information to achieve this goal. Complex or hybrid systems are characterized by frequent configuration changes, several operation modes, and the introduction or removal of sensors. This is particularly true with the Internet of Things (IoT) systems.

Hybrid dynamical systems need a combination of continuous and discrete dynamics for accurate diagnosis. Here, cutting-edge tools that can adapt to ever-evolving models and measurement needs are necessary for precise diagnoses.

Current industrial processes are transforming to become intelligent ones as Industry 4.0 becomes more popular. To detect and diagnose any issues that may arise and to maintain tabs on the general health of the operation, many modern manufacturing processes use a suite of highly sophisticated sensors. Process efficiency in today’s highly automated industrial environments requires close monitoring, process control, and timely, accurate corrective actions. Keeping an acceptable performance in industrial processes that usually contain multiple forms of defects is a serious problem.

One of the essential control techniques available for overseeing processes is failure detection and diagnosis (FDD) since most businesses want to improve their process performance by enhancing their FDD capabilities [23]. Fault detection and diagnosis (FDD) has two major purposes: number one, monitoring the condition of the procedure (the variables), and secondly, exposing the occurrence of flaws, their features, and their causes. Maintaining high production outcomes and throughput in industrial processes necessitates using fast, accurate, and effective procedures for identifying and diagnosing process or equipment faults that may threaten the presentation of the whole system.

Over several decades, numerous businesses and educational institutions have paid close attention to the FDD for a wide range of processes due to numerous considerable benefits, including decreased production-related costs and improved quality and output. In particular, FDD has been crucial in various fields of industrial engineering, including the semiconductor industry, the chemical industry, and the software business.

Thus, there is an increasing demand for reliable problem detection and diagnosis to guarantee operations function effectively and economically, avoiding expensive downtime that can affect product yield or process throughput. Most of the time, the FDD work is supervised by devices that collect data from various processes and equipment (i.e., sensors). Many current applications and research have investigated alternative analytical ways of detecting a problematic process (or variable), and many have employed different mathematical or probabilistic models to diagnose, isolate, and determine the reasons for a process abnormality after detection [6,15].

FDD approaches have been theorized and used in industry. FDD’s toolset includes data-, model-, and knowledge-based methodologies. Data-driven and model-based techniques have influenced FDD for industrial processes because of its simplicity of use. These techniques need minimal modeling and process expertise (or relevant variables).

Online, real-time FDD is another key problem in process monitoring, particularly for organizations that employ potentially harmful processes, like industrial-scale chemical processes. The FDD tool may boost productivity and security by identifying abnormalities at their early stages. However, problems in the control loops of chemical processes are notoriously difficult to pinpoint because of the masking effects of feedback. Many have proposed using a set of FDD models that can distinguish between broken and functional states to fix the problem.

Numerous successful FDD approaches have been developed due to the widespread interest in using FDD tools for process monitoring from various research and application disciplines. Each of the four phases of a general process monitoring procedure—fault detection, isolation, identification, and recovery—makes up a single loop.

Numerous specialized phrases are often used in process monitoring, and these terms must be defined and categorized in line with their unique features. An error occurs when a process variable exhibits unexpected behavior or an abrupt change in value. The gap between a threshold value and a fault value indicates an undesirable deviation, which may lead to a malfunction or a total breakdown of the process. It’s conceivable that flaws have already been incorporated into the process, and there’s no set rate at which they become evident. Disturbances are often classified as sudden (also called stepwise fault), incipient (also called drifting fault), or intermittent based on the rate at which they initially become noticeable. Defects may also be categorized according to their reflection method, with additive and multiplicative faults. A failure occurs when the system cannot complete its intended task because of an unending delay. Therefore, the failure indicates that a process unit’s role has concluded and is caused by some interrelated factors [4]. Based on their predictability, failures may be classified as random (unpredictable), deterministic, or systematic/casual. The vast majority of breakdowns occur due to random causes. When anything goes wrong, it causes a breakdown, which is an abnormal halt in the functioning of a process or system. Now we may conclude that a problem should never be ignored.

One of the most well-known methods for monitoring processes in a commercial context is statistical process control (SPC), also known as statistical process monitoring (SPM). To be used with traditional univariate SPM methods for monitoring a single process variable, the observed process variables must be independent, stable, and normally distributed (i.e., Gaussian). However, for many real-world industrial processes, this assumption is usually wrong since the underlying properties of these processes are multivariate, non-linear, non-Gaussian, and non-stationary. This drastically reduces the usefulness of conventional SPC methods. Inadequate findings may occur If a univariate control chart is used to examine a multivariate system with non-linear and cross-correlated variables. Many enhanced multivariate statistical process monitoring (MSPM) approaches have been developed to overcome the inadequacies of old univariate SPC methods for monitoring dynamic industrial processes. Useful statistics are a part of these methods. Many researchers have devoted time and effort to the problem of monitoring non-measurable process variables like status and parameters. As a consequence, process-based models and practical estimating methodologies have found widespread application in the field. In particular, there has been a lot of interest in a wide variety of effective process monitoring and fault diagnosis (PM-FD) systems built upon multivariate statistical methods since the advent of sensor technology that enables the regular collection of data on a large number of process variables [4]. Systems like this are intended to detect and isolate issues in real-time in a process. The study presented two well-known multivariate-based projection strategies for process monitoring [4]. The principal component analysis (PCA) and partial least squares (PLS) techniques are the names given to these methodologies, respectively. Both continuous and multivariate batch processes were used to demonstrate these methods.

The authors of this research elaborated on the merits of combining multi-block PLS with contribution plots. The mineral processing facilities used PCA and PLS in both their continuous and batch processes. By considering the long-term tendencies of semiconductor wafer batches, Peres et al. [15] enhanced the multivariate SPC (MSPC) method for defect detection. The authors of this work employed the log transformation approach to linearize the acquired data to analyze the long-term variability in optical emission data from a semiconductor plasma etcher. The goal was to simplify working with the information gained from this. In addition, they provided a reliable method for defect diagnosis by fusing optical emission data with sensor data and filtering the findings to account for the impact of the machine age. They had laid the groundwork for a system for defect detection that drew from several data sources and accounted for human error. A new fault detection and isolation (FDI) method for the on-board diagnosis of early faults in dynamic systems was introduced by Okwuosa et al. [2]. They also provided a detailed statistical approach for doing so.

The goal of this approach was to create a novel method of fault detection and isolation (FDI). Principles of the local approach, a mathematical and statistical theory underlying the possibility of performing preliminary FDI operations on board, were outlined in this study [2]. The researchers who found these results were responsible for another study. To maintain optimum process conditions and prevent any significant loss of system efficiency in today’s industrial environment [25], it is crucial to detect flaws as soon as possible. While multiple MSPM techniques have been used for fault detection in a wide range of industrial processes, MSPM based on conventional fault detection approaches may struggle to handle issues in their infancy. Okwuosa introduced a new incipient fault detection method predicated on a comprehensive fault detection index after discussing the six distinct fault detection indices used in conventional PCA and PLS [2]. This was conducted to launch the strategy built on a comprehensive fault detection index. The moving average (MA) and the exponentially weighted moving average (EWMA) methods were employed to determine these indices.

To enhance the usefulness of MCCs (multivariate control charts) in process monitoring, Paul et al. [16] introduced cutting-edge variable selection techniques combined with multivariate statistical process control (MSPC) methodologies. In total, 30 MSPC strategies were identified and organized into ten groups, defined by their shared objectives and process monitoring methods. Specifically, they split it into pre-processing and post-processing filter phases and a wrapper step. Among the several MSPM strategies, principal component analysis-based methods have seen widespread use for fault detection and diagnosis. Principal component analysis (PCA) is a dimensionality reduction technique that uses the data’s variance structure to quickly identify problems. Recursive principal component analysis (RPCA), dynamic principal component analysis (DPCA), and kernel principal component analysis (KPCA) are all PCA variants that have found use in the monitoring of non-linear, adaptive, and other complex industrial processes. Pinto et al. [14] introduced two RPCA methods to deal with the ongoing change in semiconductor manufacturing. They did a recursive update of the correlation matrix to reach this point. Sample-wise recursion was handled using the rank-one modification technique, while block-wise recursion was handled using the Lanczostridiagonalization technique.

Qi et al. [16] voiced worry that DPCA decompositions were not sensitive to early process faults, which typically affect the covariance structure of variables and the underlying DPCA decomposition. The MSPC plan was amended to include a regional strategy, and the goal was achieved. They also developed a 3D fault diagnosis chart to display potential shifts brought on by a malfunction. Using this method, they could zero in on the most crucial aspects of the process that may be to blame for the issues. Qi generalized and analyzed five distinct fault diagnosis techniques in [16]. After demonstrating that the five methods may be combined into three general ones, they provided the predicted contributions and the strategies’ relative contributions. They found that different diagnosis techniques may not provide a proper diagnosis for naïve sensor errors of low magnitude, based on their study on the diagnosis of process failures. The presented fault diagnosis algorithms were assessed by Monte Carlo simulation. By estimating non-linearity in processes, Schimmack and Mercorelli [7] have developed a real-time fast block AKPCA-based variable window monitoring system that can detect and respond to normal drifts by adjusting the parameters and size of adaptive charts. Researchers showed the model’s resilience and improved detectability of process deviations by using it to monitor two conventional processes.

Once a problem has been identified, the following step is often to isolate the offending process or variable to learn more about the issue, such as what led to the failure. Fault diagnostics involves learning as much as possible about the problem at hand, including how it came to be, where it originated, when it was first observed, and what effects it has. It is common to think of fault diagnosis as a multi-stage process that includes detection, isolation, identification, classification, and assessment. Today, several industries make use of the proven FDD techniques. For this reason, many researchers have delved into the topic of FDD, using methods like multivariate analysis, analytical methods, artificial intelligence, etc., as proposed by Mercoelli [59]. In particular, several useful ways have been developed and used for dealing with various types of FDI difficulties, including hierarchical methodologies. Data-driven solutions for spotting and removing problematic variables mostly fall into two categories: the supervised approach and the unsupervised approach. In the supervised approach, the fault subspace or area of abnormal operation for each faulty state must be defined a priori; in the unsupervised approach, however, the faults may be separated using just a priori knowledge, such as the contribution plot of faulty variables from measured data.

However, if the a priori information on error-prone occurrences is unknown, the supervised strategy may not be effective. The FDI method consists of two primary stages: residual generation and evaluation. To isolate the process parameter change that leads to the observed structural damages, a locally focused approach and two straightforward statistical tests, such as sensitivity analysis and a min–max test-based residual evaluation method, are offered. To illustrate the efficacy of the developed technique, two example case studies of vibration-based structural damage diagnoses were presented. Fault detection is difficult because of the complexity of many industrial processes, which can include several process variables, quantifiable data that is contaminated or cross-correlated, and intricate links between symptoms and issues. In response, several useful methods for fault identification have been developed to record the characteristics of the flaws.

Daga et al. [60] also discussed the basic problems and methods for monitoring processes, such as FDD, which may be applied to specific technical operations. In this study, the authors introduced a knowledge-based technique and a suite of defect detection algorithms that draw on process and signal models to extract targeted features from collected data. They created analytic symptoms and heuristic symptoms, often caused by human operators, as an additional source of information to compare the behaviors of the process (i.e., normal and aberrant behaviors). They also introduced fuzzy algorithms, classification systems, and approximate reasoning based on if-then rules. Many different processes in the chemical industry may benefit from using FDD since it is such an effective process monitoring tool. To guarantee safe and efficient operations, the chemical industry requires state-of-the-art FDD methods for detecting and analyzing process flaws. Among the several FDD methods available, principal component analysis (PCA) is widely used for identifying outliers in chemical procedures. Fault detection and diagnostics (FDD) in chemical processes are also acknowledged as an integral part of the abnormal event management (AEM) system, which includes the three essential duties of fault detection, fault diagnosis, and corrective action for the defects in a process. But it is well-known that many researchers are working on overcoming many practical obstacles in real chemical processes, such as how to cope with non-linearity, non-stationarity, autocorrelation, cross-correlation, non-Gaussian distribution, etc., rather than on applications of FDD. Note that most genuine chemical process data has multi-scale features. In other words, real-world data from chemical processes have a lot of time- and frequency-dependent features and noise.

Some crucial elements are frequently hidden since undiscovered inaccuracies may contaminate the measured data. Methods for troubleshooting two different chemical processes were examined. After evaluating the prediction error of the NN models for sensor or component failure detection, the root cause of the issue was identified using a radial basis function (RBF) neural classification strategy. Two representative chemical processes were used to evaluate the neutral network (NN) models. Okwuosa [2] introduced a subspace strategy for developing efficient FDII (fault detection, isolation, and identification) systems. In this study, four methods were presented for producing FDII residuals.

The lateral dynamic system of the vehicle was analyzed virtually to show that the proposed algorithms are robust against inputs while being sensitive to faulty sensors and actuators. Using MCCA (multiset canonical correlation analysis), Okwuosa [2] presented a unique joint–individual process monitoring technique for chemical processes. In this study, the researchers utilized MCCA to identify common process characteristics and projected data from a single operational unit in terms of joint and individual attributes. Finally, they created statistics to seek commonalities across the two levels of analysis. Waghen et al. [61] reflected on the pros and cons of using the FDD techniques in chemical processes. They investigated typical problems encountered in actual chemical processes and looked at how big data analytics and AI-based approaches may provide solutions. To improve production efficiency in the semiconductor industries, more process and quality management methods and process automation are required. Developing a solid FDC system that can be used in the semiconductor industry is becoming more important. It is essential to maintain regular and secure production by minimizing or doing away with any irregular variation seen in semiconductor manufacturing processes via prompt and precise issue diagnostics.

Accordingly, fault detection and classification (FDC) in semiconductor manufacturing is now generally accepted as an integral element of the APC (advanced process control) framework for maximizing production efficiency (yield and machine utilization) while minimizing process variation [18]. The semiconductor industry has benefited from developing and implementing many FDC techniques that aid in issue identification and root cause investigation.

In their analysis, Wang et al. [3], to solve this problem, used fault diagnosis (PM-FD) and KPI-based process monitoring schemes to identify deficient process variables and their root causes from the viewpoint of performance decline. Many examples were provided to prove the method’s validity and usefulness. Wang et al. [3] looked at a problem with defect detection in non-linear systems, as shown by the Takagi–Sugeno model. The authors of this study combined the influence of disturbances with residual generators to amplify the fault effects while dampening perturbation ones. Thus, Wang et al. [3] suggested a unique hybrid FD approach for non-linear systems. This was accomplished using a mathematical model and some ingenious AI techniques. They created a neural parameter estimator (NPE) based on a single-parameter fault model, two NPE structures, and updating techniques for FDI weights and decision logic. The team also implemented a fault-tolerant observer to better predict future system states. Fault diagnosis may be broken down into four categories, as provided by Waghen et al. [61]: unknown faults, known faults, multiple dependent faults, and multiple independent faults. They achieved this by integrating features based on the information’s path with those derived from two ways (a modified distance-based approach and a modified causal dependency-based approach). Xiao adjusted a Gaussian kernel for defect identification using five evaluation criteria and two data preparation techniques (KFDA and KPCA).

Using Tennessee Eastman benchmark process data, an ANN model was verified and compared to the KFDA and KPCA. Bindi employs an NLGBN model to detect manufacturing line difficulties [20]. They devised a three-layer NLGBN model for feature extraction from noisy data, using sigmoidal functions to find non-linear correlations between process and latent variables. Xio presented two evidence-based decision fusion systems. First, they employed resampling to boost diversity performance, then “ALL fusion” and “SELECTIVE fusion” for defect detection and classification. Xio studied fault detection in chemical processes. They employed circuit card assembly (CCA) and a moving window to find multiplicative initial faults. Xio identified defects despite class imbalance. The researchers analyzed 19 fault detection algorithms utilizing data from two semiconductor manufacturing processes. Yang et al. [62] developed a method for identifying process defects. They applied Granger causality analysis and presented a causality score based on the DTW (dynamic time warping) approach.

Yang et al. [62] studied FDI input signal design. This study offered a graph–theory based multi-parametric programming strategy to minimize the FDI complexity and increase the computing efficiency. Yang et al. [62] created a unique way of building the FDD framework, making it adaptable to various complexity, noise, and dimensionality concerns. The authors employed wavelet analysis, kernel discriminant analysis, and support vector machine classifiers to discover chemical process defects. The Tennessee Eastman benchmark shows that the combined strategies work. Ferentinos et al. presented a data-driven fault detection method based on recursive transformed component analysis to discover process faults quickly [20]. To decrease computational complexity, they changed the process data using rank-one modification—a detection index—which Ferentinos et al. developed for identifying process characteristics. Ferentinos et al. introduced a PM-FD framework based on KPIs to enhance the monitoring of large-scale industrial operations. When there is a static statistical correlation between process variables and KPIs, they use a static method. MSPC approaches were sensitive to mild changes in the process variables.

This study established a new FDD approach to improve early fault isolation (the data covariance structure). First, they utilized sparse regression to detect problematic process variables due to a data distribution structure change. Then, they used distribution dissimilarity decomposition to uncover differences between problematic and normal process circumstances. Yang et al. [62] studied failure detection using connected process data. They invented dynamic graph embedding to secure the process variables and data, such as the structural information in process variables and serial (or temporal) relationships among process data digital gene expression (DGE). Later, they updated similarity matrices based on the finite Markov chain to highlight essential process aspects. To illustrate the method’s feasibility, they used the Tennessee Eastman benchmark. Ferentinos et al. [20] studied non-linearity in semiconductor etching to find defects.

Researchers used multiway principal polynomial analysis to identify faults. They used a numerical example and semiconductor etching data to verify their technique worked. Achieving full fault tolerance of the neural network (the absence of a negative reaction of the network to defects arising in it) is possible in the case of at least a six-fold redundancy of neurons [53], which is not always acceptable due to the technical complexity of the hardware implementation. In the absence of redundancy, as noted by the authors of [63], almost any defect always affects the completeness and quality of the performance of functions since all its components are involved in neural network calculations.

The widespread use of neural networks in various fields of technology necessitates the development of methods for improving their reliability and corresponding estimates of reliability characteristics. The study of the reliability of neural networks and the degree of their gradual degradation is almost impossible without modeling defects in neuro components [41]. The faults considered in the studies are models of defects that determine the place, time, and nature of their manifestation.

One of the shortcomings of the existing defect models is that they take into account only the weight coefficients set to zero states as defects and the maximum or minimum signal values at the outputs of neurons. In reality, physical defects manifest themselves in a much more complex way and can often lead to several malfunctions of a neuron.

## 4. Conclusions

Numerous process monitoring systems, including the FDD methods, have been explored and implemented for various industrial processes due to the widespread interest in using the FDD approaches for effective process monitoring. However, many challenges remain in applying the FDD techniques to actual industrial processes because of their specific nature (e.g., multivariate, correlation, non-linearity, non-stationarity, multimodality, class imbalance, etc.). Given this somewhat wide gap between theoretical methods and implementations, it is important to examine fresh hybrid approaches and create more intricate FDD models by employing various intelligent strategies. Future studies will also need to focus on improving fault prognosis. It is possible to avert some safety issues in advance and stop the further deterioration and destruction of goods if the location and time of faults developing in the processes can be anticipated or predicted adequately.

Last but not least, in the age of big data, real-time, and comprehensive FDD strategies, using all the information should be established. As a result, this study will be useful for scholars and practitioners alike in understanding the essential features, practical uses, and impending difficulties of process monitoring and FDD methods.

## Data Availability

Not applicable.
